# Relationship between digital exclusion and cognitive impairment in Chinese adults

**DOI:** 10.3389/fnagi.2023.1194348

**Published:** 2023-07-03

**Authors:** Xiaoli Liu, Xiaoxiao Wang, Hua Zhang, Minyue Pei, Nan Li

**Affiliations:** ^1^Research Center of Clinical Epidemiology, Peking University Third Hospital, Beijing, China; ^2^Department of Hernia and Abdominal Wall Surgery, Beijing Chaoyang Hospital, Capital Medical University, Beijing, China

**Keywords:** cognitive impairment, digital exclusion, cross-sectional study, CHARLS, China

## Abstract

**Objective:**

We aimed to evaluate the relationship between digital exclusion, such as neither mobile payments nor WeChat use, and cognitive impairment in Chinese individuals aged 45 and older.

**Methods:**

A population-based cross-sectional study utilizing data from the fourth national survey of the China Health and Retirement Longitudinal Study (CHARLS). In the fourth wave of CHARLS, 10,325 participants aged 45 and older with complete information were included in this analysis. Self-reported mobile payments and WeChat usage constituted our exposure. Cognitive impairment was the primary outcome. Univariate and multivariate logistic regression were used to assess the relationships between cognitive impairment risk and digital exclusion.

**Results:**

Data were analyzed from 10,325 participants [mean (SD) age, 60.3 (9.1) years; 44.8% women], including 1,232 individuals with cognitive impairment and 9,093 cognitively normal individuals. The overall proportion of users who did not use either mobile payment or WeChat and those who only used WeChat were 81.3 and 6.7%, for cognitively impaired individuals 95.0 and 3.1%, and for cognitively normal individuals 79.5 and 7.2% [neither WeChat nor mobile payments vs. control unadjusted odds ratio (OR), 8.16; *P* < 0.001; only WeChat use vs. control unadjusted OR, 2.91; *P* < 0.001]. Participants who did not use either WeChat or mobile payments had an elevated risk for cognitive impairment after adjusting for a number of covariates (neither WeChat nor mobile payments vs. control adjusted OR, 3.48; *P* < 0.001; only WeChat use vs. control adjusted OR, 1.86; *P* = 0.021).

**Conclusion:**

Our study reveals a positive correlation between digital exclusion and cognitive impairment in Chinese adults, providing insights for promoting active digital integration among older adults. Further longitudinal research is needed to further validate this hypothesis.

## Introduction

Cognitive impairment can be a devastating and disabling disease that places a substantial burden on patients, families and society, and has become a serious global concern in most developed and developing countries due to population aging. The global prevalence of dementia, which is a type of cognitive impairment, was estimated to be 46 million in 2015, with the number expected to triple by 2050 (Global Burden of Disease Study 2013 Collaborators, 2015). A recent meta-analysis conducted by Zhu et al. found that the pooled prevalence of dementia worldwide among the elderly aged 60 years and older was 4.86% (95% CI: 4.20–5.63%) (Zhu et al., 2019). China has the largest number of dementia patients, placing a significant strain on the public and healthcare system (Liu et al., 2013). The prevalence of dementia was 5.14% (95% CI 4.71–5.57%) in 2014 and 5.60% (3.50–7.60%) in 2019 among those aged 65 and older, in China (Jia et al., 2014; Huang et al., 2019). With China’s aging population on the rise, the prevalence of cognitive impairment is also on the rise (Jia et al., 2020). Understanding the mechanism of cognitive impairment and developing effective preventative and therapeutic methods necessitates a thorough examination of factors that contribute to its development.

Digital exclusion, defined as the inequity in access and capability to use information and communications technologies (ICTs) such as the internet, may be associated with cognitive impairment, although the relationship is complex and not fully understood (Seifert, 2020; Wilson et al., 2022). On the one hand, the use of digital technologies such as WeChat, mobile payments or the Internet has been shown to have cognitive benefits, such as improved memory, attention, and problem-solving skills (Radić et al., 2021; Wei et al., 2022). Thus, individuals with a lack of digital technology or limited digital skills may miss out on these cognitive benefits, which may lead to cognitive impairment over time. On the other hand, cognitive impairment itself can be a barrier to digital inclusion, as it can make it more difficult for individuals to learn new digital skills and use digital technologies effectively (Anderberg et al., 2019; Gerritzen et al., 2023). For example, people with cognitive impairment may have difficulty dealing with complex interfaces or have difficulty remembering passwords or following online instructions (Cunnah et al., 2021). Overall, while there is some evidence for a relationship between digital exclusion and cognitive impairment, further research is needed to better understand the complex interplay between digital exclusion and cognitive impairment.

The China Health and Retirement Longitudinal Study (CHARLS) is a large-scale, multiwave study of middle-to-old aged individuals that is representative of the country’s population (Zhao et al., 2014). This study made it possible to examine the relationship between cognitive function and digital exclusion like not using WeChat or mobile payments. Therefore, by analyzing the data from CHARLS, we intended to assess the relationship between cognitive function and digital exclusion in the present study. We hypothesized that not using WeChat or mobile payments would have a positive correlation with cognitive impairment in Chinese adults.

## Materials and methods

### Study population

**FIGURE 1:**
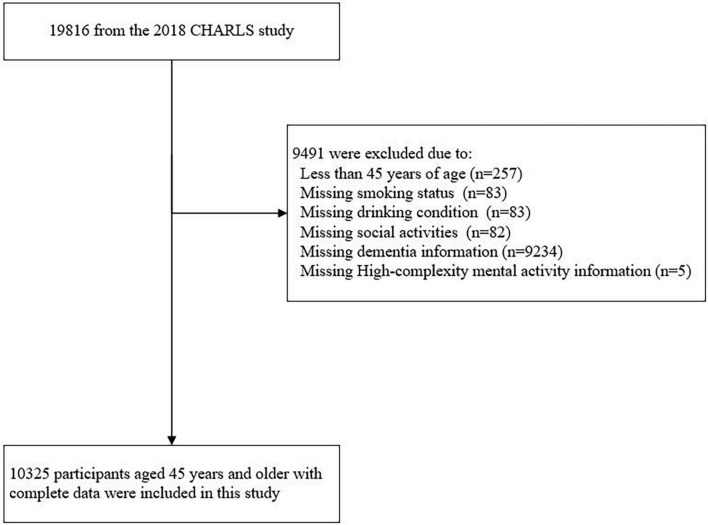
Flow chart of enrollment of study subjects.

The CHARLS is a nationally representative longitudinal survey of Chinese persons aged 45 and over, assessing the social, economic, and health status of community members. The fourth national survey was conducted between July 2018 and March 2019, among 19,816 participants. Because mini mental state exam (MMSE) and digital exclusion were collected in the fourth wave of CHARLS for the first time, data from wave 4 (2018) of the CHARLS were used in the present study. This study included data from 10,325 participants with complete data (Figure 1).

The CHARLS was approved by (Anonymized for Review). Written informed consent was obtained from each participant in accordance with the Declaration of Helsinki. Deidentified data from the CHARLS were used in this analysis. This study followed the Strengthening the Reporting of Observational Studies in Epidemiology (STROBE) reporting guideline.

### Digital exclusion

Data on digital exclusion were collected through self-completed questionnaires. In the CHARLS, digital exclusion was assessed using the questions “Do you use mobile payments, such as Alipay and WeChat pay?” and “Do you use WeChat?” In the data analysis phase, participants were classified into three categories based on the participant’s response: using WeChat and mobile payments, only using WeChat, and neither (digital exclusion).

### Cognitive assessment and definition of cognitive impairment

The CHARLS assessed the cognitive function of participants using the Chinese version of the MMSE, with excellent validity and reliability (Xu et al., 2018; Arevalo-Rodriguez et al., 2021). The MMSE consists of 30 items. The MMSE score ranged from 0 to 30 and higher scores indicate better cognitive function. We defined cognitive impairment as a MMSE score lower than 17 for an illiterate level individual, lower than 20 for individuals with elementary school, and lower than 24 for individuals with middle school and higher (Zhang, 1990).

### Covariates

Covariates that might confound the association between digital exclusion and cognitive impairment in the analyses included sex, age (in years), level of education, cohabitation status, smoking status, drinking condition, physical activities, social activities, hypertension, diabetes, dyslipidemia, stroke, chronic lung diseases, memory-related disease, hearing disorder, and emotional, nervous, or psychiatric problems. All covariates were obtained from the CHARLS database (Zhao et al., 2020).

### Statistical analysis

Variables of a categorical nature were expressed as frequencies and percentages. Continuous variables were characterized by their mean value and standard deviation (SD). The Chi-square test or independent sample *t*-test was performed to determine whether there were statistically significant differences between normal individuals and those with cognitive impairment regarding digital exclusion and other influencing factors. Using binary logistic regression, the relationships between cognitive impairment risk and digital exclusion were assessed. The odds ratios (ORs) and 95% confidence intervals (CIs) were computed to quantify the effects of digital exclusion.

We assessed the robustness of the association between digital exclusion and cognitive impairment by including different confounding covariates in different models. Model 1 did not adjust for any variables; Model 2 adjusted for age, sex, education level, smoking status and drinking condition; Model 3 added cohabitation status, physical activities, and social activities; Model 4 additional adjusted for hypertension, diabetes, dyslipidemia, stroke, chronic lung diseases, emotional, nervous, or psychiatric problems, memory-related disease and hearing disorder (the fully adjusted model).

Regarding that older individuals had a positive correlation with cognitive impairment, and were less likely to use mobile payments and WeChat, associations between digital exclusion and cognitive impairment were examined in the sub-population, respectively (< 80 years old, < 70 years old) as sensitivity analyses. A sensitivity analysis was also conducted according to residential status (urban, transitional zone, rural).

Data analysis was performed using StataSE 15 (StataCorp, College Station, TX, USA). If the *P*-value was less than 0.05, it was considered statistically significant.

## Results

### Characteristics of the participants

In the fourth wave of CHARLS in 2018, 19,816 individuals participated in the survey, 10,325 participants had a complete dataset (Figure 1), which consisted of 1,232 individuals with cognitive impairment and 9,093 cognitively normal individuals. Of 10,325 individuals, which were included in this analysis, the mean (SD) age was 60.3 (9.1) years; 44.8% (4,627 of 10,325) were women; 12.0% (1,233 of 10,325) and 6.7% (696 of 10,325) used mobile payments, and WeChat, respectively.

Compared with cognitively normal individuals, individuals with cognitive impairment had a significantly lower proportion of WeChat and mobile payments use. For cognitively normal individuals, the proportions of users who did not use either mobile payment or WeChat and those who only used WeChat were 79.5 and 7.2%, respectively, while for cognitively impaired individuals, the proportions were 95.0 and 3.1%, respectively (Neither WeChat nor mobile payments vs. control unadjusted OR, 8.16; 95% CI, 5.42 to 12.28; *P* < 0.001; only WeChat use vs. control unadjusted OR, 2.91; 95% CI, 1.73 to 4.90; *P* < 0.001). In addition, the study subjects with cognitive impairment were older, and were more likely to live in rural areas than the cognitively normal population. The characteristics of the participants are shown in Table 1.

**TABLE 1:** Characteristics of the study participants.

Characteristic	Cognitively normal (*n* = 9,093)	Cognitively impaired (*n* = 1,232)	Total (*N* = 10,325)	*t*/χ ^2^	*P*
Digital exclusion				176.974	< 0.001
Neither WeChat nor mobile payments	7,221 (79.46%)	1,170 (94.97%)	8,391 (81.31%)		
Only WeChat	658 (7.24%)	38 (3.08%)	696 (6.74%)		
Use WeChat and mobile payments	1,209 (13.3%)	24 (1.95%)	1,233 (11.95%)		
Age	59.38 ± 8.92	67.27 ± 6.72	60.32 ± 9.06	−29.910	< 0.001
Sex				2.539	0.111
Female	4,101 (45.1%)	526 (42.69%)	4,627 (44.81%)		
Male	4,992 (54.9%)	706 (57.31%)	5,698 (55.19%)		
Education				29.875	< 0.001
Junior school and below	1,786 (19.64%)	162 (13.15%)	1,948 (18.87%)		
High school and above	7,307 (80.36%)	1,070 (86.85%)	8,377 (81.13%)		
Smoking status				19.155	< 0.001
Still have	2,722 (29.96%)	395 (32.14%)	3,117 (30.22%)		
Quit	1,429 (15.73%)	241 (19.61%)	1,670 (16.19%)		
Never smoked	4,933 (54.3%)	593 (48.25%)	5,526 (53.58%)		
Drinking condition				18.616	< 0.001
Drink more than once a month	2,879 (31.69%)	343 (27.91%)	3,222 (31.24%)		
Drink but less than once a month	836 (9.2%)	84 (6.83%)	920 (8.92%)		
None of these	5,369 (59.1%)	802 (65.26%)	6,171 (59.84%)		
Living alone				96.394	< 0.001
Yes	853 (9.38%)	228 (18.51%)	1,081 (10.47%)		
No	8,240 (90.62%)	1,004 (81.49%)	9,244 (89.53%)		
Physical activities				21.706	< 0.001
Yes	8,551 (94.04%)	1,116 (90.58%)	9,667 (93.63%)		
No	542 (5.96%)	116 (9.42%)	658 (6.37%)		
Social activities				73.246	< 0.001
Yes	5,718 (62.95%)	618 (50.28%)	6,336 (61.44%)		
No	3,366 (37.05%)	611 (49.72%)	3,977 (38.56%)		
Hypertension				3.070	0.080
Yes	916 (10.07%)	144 (11.69%)	1,060 (10.27%)		
No	8,177 (89.93%)	1,088 (88.31%)	9,265 (89.73%)		
Diabetes				0.017	0.895
Yes	457 (5.03%)	63 (5.11%)	520 (5.04%)		
No	8,636 (94.97%)	1,169 (94.89%)	9,805 (94.96%)		
Dyslipidemia				0.001	0.977
Yes	925 (10.17%)	125 (10.15%)	1,050 (10.17%)		
No	8,168 (89.83%)	1,107 (89.85%)	9,275 (89.83%)		
Stroke				27.468	< 0.001
Yes	356 (3.92%)	88 (7.14%)	444 (4.3%)		
No	8,737 (96.08%)	1,144 (92.86%)	9,881 (95.7%)		
Chronic lung diseases				2.246	0.134
Yes	428 (4.71%)	70 (5.68%)	498 (4.82%)		
No	8,665 (95.29%)	1,162 (94.32%)	9,827 (95.18%)		
Emotional, nervous, or psychiatric problems				0.654	0.419
Yes	71 (0.78%)	7 (0.57%)	78 (0.76%)		
No	9,022 (99.22%)	1,225 (99.43%)	10,247 (99.24%)		
Memory-related disease				21.515	< 0.001
Yes	113 (1.24%)	36 (2.92%)	149 (1.44%)		
No	8,980 (98.76%)	1,196 (97.08%)	10,176 (98.56%)		
Hearing disorder				41.554	< 0.001
Yes	852 (9.37%)	188 (15.26%)	1,040 (10.07%)		
No	8,241 (90.63%)	1,044 (84.74%)	9,285 (89.93%)		
Residential status				80.703	< 0.001
Urban	2,323 (25.55%)	214 (17.37%)	2,537 (24.57%)		
Transitional zone	933 (10.26%)	73 (5.93%)	1,006 (9.74%)		
Rural	5,791 (63.69%)	943 (76.54%)	6,734 (65.22%)		
Special area	46 (0.51%)	2 (0.16%)	48 (0.46%)		

### Logistic regression analysis of the factors associated with cognitive impairment

The results showed that neither WeChat nor mobile payment use was associated with poorer cognitive function (Table 2). Participants who did not use either WeChat or mobile payments had a positive correlation with cognitive impairment after adjusting for a number of covariates (Neither WeChat nor mobile payments vs. control adjusted OR, 3.48; 95% CI, 2.27 to 5.33; *P* < 0.001; only WeChat use vs. control adjusted OR, 1.86; 95% CI, 1.10 to 3.16; *P* = 0.021). In the fully adjusted model, we also found that being older (OR 1.09, 95% CI 1.08–1.10), still smoking (OR 1.43, 95% CI 1.17–1.74), and having a stroke (OR 1.32, 95% CI 1.02–1.70) had a positive correlation with cognitive impairment. Male (OR 0.79, 95% CI 0.65–0.96) and individuals participating in social activities (OR 0.83, 95% CI 0.73–0.95) had a negative correlation with cognitive impairment (see Supplementary Table 1 for details).

**TABLE 2:** Associations between using WeChat, mobile payments and cognitive impairment in the overall population.

Variables	Model 1		Model 2		Model 3		Model 4	
	**OR (95% CI)**	***P***	**OR (95% CI)**	***P***	**OR (95% CI)**	***P***	**OR (95% CI)**	***P***
**Digital exclusion**								
Neither WeChat nor mobile payments	8.16 (5.42, 12.28)	< 0.001	3.76 (2.47, 5.74)	< 0.001	3.45 (2.25, 5.29)	< 0.001	3.48 (2.27, 5.33)	< 0.001
Only WeChat	2.91 (1.73, 4.90)	< 0.001	1.84 (1.09, 3.12)	0.023	1.86 (1.09, 3.15)	0.022	1.86 (1.10, 3.16)	0.021
Use WeChat and mobile payments	Ref.	–	Ref.	–	Ref.	–	Ref.	–

Model 1: No variables are adjusted. Model 2: Adjusted for age, sex, education level, smoking status and drinking condition. Model 3: Adjusted for age, sex, education level, smoking status, drinking condition, living alone, physical activities, and social activities. Model 4: Adjusted for age, sex, education level, smoking status, drinking condition, living alone, physical activities, social activities, hypertension, diabetes, dyslipidemia, stroke, chronic lung diseases, emotional, nervous, or psychiatric problems, memory-related disease, hearing disorder.

### Sensitivity analysis

**FIGURE 2:**
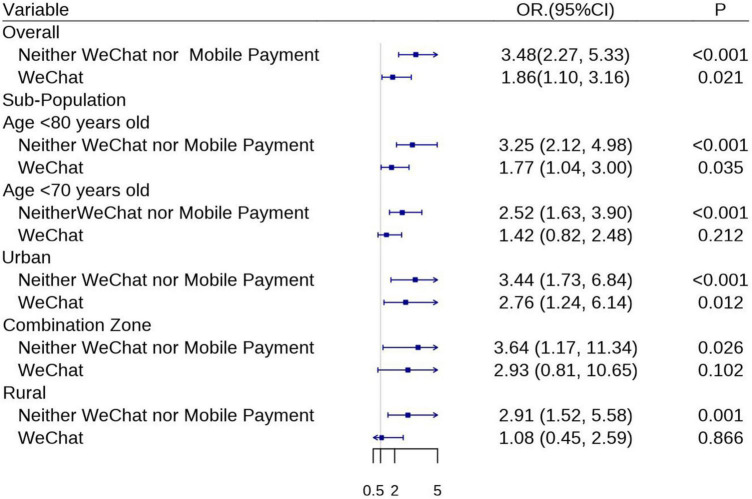
Associations between using WeChat, mobile payments and cognitive impairment, in the overall population and sub-population. Presented with adjusted odds ratio (OR) and 95% confidence intervals (CIs) when adjusting for age, sex, education level, smoking status, drinking condition, living alone, physical activities, social activities, hypertension, diabetes, dyslipidemia, stroke, chronic lung diseases, emotional, nervous, or psychiatric problems, memory-related disease, and hearing disorder.

Considering that age and residence status may influence the association between digital exclusion and cognitive impairment, sensitivity analyses were conducted. Mobile payments and WeChat use, remained protective against cognitive impairment when we excluded individuals aged 80 and over, or when we excluded individuals aged 70 and over (In those aged < 80: neither WeChat nor mobile payments vs. control adjusted OR 3.25, 95% CI 2.21–4.98; only WeChat use vs. control adjusted OR 1.77, 95% CI 1.04–3.00; In those aged < 70: neither WeChat nor mobile payments vs. control adjusted OR 2.52, 95% CI 1.63–3.90; only WeChat use vs. control adjusted OR 1.42, 95% CI 0.82–2.48; Figure 2 and Supplementary Table 1).

In the stratified analysis based on the type of residential area (Figure 2 and Supplementary Table 2), people who did not use either WeChat or mobile payments had a positive correlation with cognitive impairment irrespective the type of residential area. People who only used WeChat had a positive correlation with cognitive impairment in the urban area (OR 3.44, 95% CI 1.73–6.84), but not in the rural area (OR 2.91, 95% CI 1.52–5.58), or the transitional zone located between urban and rural areas (OR 3.64, 95% CI 1.17–11.34).

## Discussion

In this large community-based study, after controlling for other confounding variables, we discovered that digital exclusion had a positive correlation with cognitive impairment. Moreover, older age, smoking, and stroke also had a positive correlation with cognitive impairment, although participating in social activities had a negative correlation with cognitive impairment.

Our data suggested that digital exclusion that is, participants who do not use either mobile payments or WeChat may have a positive correlation with cognitive impairment. Mobile payments relate to any online transaction that is explicitly initiated, granted, registered, and validated via mobile terminal equipment (Lin et al., 2022; Rehman et al., 2022). Regarding mobile terminal equipment, mobile payments relate to the acquisition and payment of products and services utilizing wireless communication technology on a cell phone, smartphone, or personal digital assistant (Singh et al., 2020). In addition to messaging, people on WeChat can also discover new content by subscribing to feeds or sharing “Moments.” Consequently, WeChat and mobile payment increase social interaction and improve cognitive stimulation. The digital exclusion of not using WeChat and mobile payments partly leads to social isolation and reduced cognitive stimulation, which is associated with cognitive decline (Kim and Han, 2022). Even when controlling for education, occupation, baseline cognition, and cardiovascular risk factors, protective effects remain significant in cohort studies of older adults that have focused on cognitive complexity in their current lifestyle (Fabrigoule et al., 1995; Fratiglioni et al., 2000; Wang et al., 2002; Wilson et al., 2002). Overall, the growth of mobile payments and WeChat has altered human lives and social habits, made daily life more convenient, and increased access to information. Examining the relationship between the usage of mobile payments and WeChat and cognitive impairment, provides helpful insights into how cognitive health of people, particularly the elderly, might be improved. This study suggests that not using social media such as WeChat and mobile payment may have a positive correlation with cognitive impairment; hence, it is vital to promote social media among the public, particularly the elderly, and to assist them bridge the digital gap. Consequently, appropriate education programs should be established to assist people, particularly the elderly, in mastering the procedures and abilities of social media use. We can also promote health education through Official WeChat account, and all these measures aim to increase cognitive function and decrease the risk of cognitive impairment.

Our observations of a relationship between age and cognitive impairment are consistent with the findings of other studies, which indicated that age had a positive correlation with cognitive impairment (Podcasy and Epperson, 2016; Lucca et al., 2020; Dominguez et al., 2021; Stute et al., 2021). Age, an immutable factor, has been identified as one of the most significant correlates with dementia (Podcasy and Epperson, 2016). A population-based study in Italy also showed a positive correlation between age and dementia, with this correlation persisting into very old age (Lucca et al., 2020). Furthermore, subjective age has been associated with cognitive decline and dementia in middle-aged and elderly adults (Stephan et al., 2017; Qiao et al., 2021). As the global population ages, cognitive deterioration and its most severe expression, dementia, inevitably increase (Prince et al., 2015; Beard et al., 2016). Currently, no treatments significantly influence dementia’s progression, with degenerative brain changes often preceding clinical symptoms (Power et al., 2018). Non-pharmacological interventions, such as good nutrition, cognitive training, and physical activity, have shown a positive correlation with various cognitive outcomes and could be pivotal in maintaining cognitive health and delaying cognitive decline (Sofi et al., 2010; Psaltopoulou et al., 2013; Singh et al., 2014; Cooper et al., 2015; Coelho-Júnior et al., 2021).

In our investigation, we discovered a positive correlation between smoking and cognitive impairment, aligning with previous studies (Pinkston et al., 2009; Singh et al., 2014; Cooper et al., 2015; Coelho-Júnior et al., 2021). Long-term smokers showed a higher correlation with cognitive impairment development than non-smokers. Moreover, environmental tobacco smoke exposure was positively correlated with severe dementia (Pinkston et al., 2009). Consequently, quitting smoking could have beneficial impacts on individual health and those around them. Concurrently, our research found a positive correlation between stroke and cognitive impairment, consistent with numerous studies (Kuiper et al., 2015; Livingston et al., 2020; Spence et al., 2020). Stroke, a significant modifiable factor, correlated strongly with all-cause dementia (Pinkston et al., 2009). Despite many stroke survivors showing significant recovery, a considerable proportion still experienced dementia (Pinkston et al., 2009). Therefore, stroke prevention strategies, including lipid-lowering therapy, a plant-based diet, appropriate blood pressure control, and B vitamins for homocysteine lowering, could significantly decrease the growing burden of stroke and dementia (Spence et al., 2020).

Moreover, our study demonstrated a negative correlation between participating in social activities and cognitive impairment, aligning with previous findings (Kuiper et al., 2015). Proposed mechanisms suggest participation in social activities could contribute to cognitive reserve and delay the clinical progression of dementia (Stern, 2002; Akbaraly et al., 2009). They may promote cognitive performance, increase brain volume, and alter cerebral plasticity, thereby correlating with a lower incidence of dementia (Jia et al., 2022; Sachdev, 2022). However, these benefits might diminish if activities are not maintained, as indicated by other studies (Richards et al., 2003; Yu et al., 2009).

We conducted a sensitivity analysis since factors such as age and residence status may impact the association between digital exclusion and cognitive impairment. For age stratification, considering advanced age, such as above 70 and 80, persons who are at high risk for cognitive impairment and who may have high levels of digital exclusion due to cognitive impairment, which will result in a reverse causal association. Consequently, we conducted a sensitivity study on populations aged < 80 and < 70, the robust results further suggested that digital exclusion may have a positive correlation with cognitive impairment.

The most significant strength of this study is that the relationship between digital exclusion, such as WeChat and mobile payments use, and cognitive impairment was examined in a large survey, for the first time to our best knowledge. Moreover, the large sample size enhanced the reliability of our statistical analysis. Nevertheless, our study has several limitations. First, because this was an observational cross-sectional study, no causal relationships could be established. Second, despite accounting for a variety of confounding factors, unmeasured covariates may have caused confounding bias. Thirdly, using self-administered questionnaires to collect data on smoking, alcohol consumption, physical activity, and other behavioral risk variables may result in incorrect estimates.

## Conclusion

This study demonstrates that digital exclusion has a positive correlation with cognitive impairment, providing a reference for promoting active integration of older adults into digital life. Longitudinal studies are needed to further test this hypothesis.

## Data availability statement

The datasets presented in this study can be found in online repositories. The names of the repository/repositories and accession number(s) can be found below: the datasets for this study can be found in the China Health and Retirement Longitudinal Study (CHARLS). The current study is a secondary analysis of public data of CHARLS. The original datasets of CHARLS is accessible on http://charls.pku.edu.cn/en.

## Ethics statement

The studies involving human participants were reviewed and approved by the Peking University Institutional Review Board. The patients/participants provided their written informed consent to participate in this study.

## Author contributions

NL had full access to all of the data in the study and took responsibility for the integrity of the data and the accuracy of the data analysis. XL, XW, HZ, MP, and NL: acquisition, analysis, interpretation of data, critical revision of the manuscript for important intellectual content, and study supervision. XL and XW drafted the manuscript. XL, XW, and HZ: statistical analysis. XL, XW, and NL obtained funding. NL: administrative, technical, or material support. All authors contributed to the article and approved the submitted version.

## References

[B1] Akbaraly T. Portet F. Fustinoni S. Dartigues J. Artero S. Rouaud O. (2009). Leisure activities and the risk of dementia in the elderly: results from the Three-City Study. *Neurology* 73 854–861. 10.1212/WNL.0b013e3181b7849b 19752452

[B2] Anderberg P. Barnestein-Fonseca P. Guzman-Parra J. Garolera M. Quintana M. Mayoral-Cleries F. (2019). The effects of the digital platform Support Monitoring and Reminder Technology for Mild Dementia (SMART4MD) for people with mild cognitive impairment and their informal carers: protocol for a pilot randomized controlled trial. *JMIR Res. Protoc.* 8:e13711. 10.2196/13711 31228177 PMC6611150

[B3] Arevalo-Rodriguez I. Smailagic N. Roque-Figuls M. Figuls M. Ciapponi A. Sanchez-Perez E. (2021). Mini-Mental State Examination (MMSE) for the early detection of dementia in people with mild cognitive impairment (MCI). *Cochrane Database Syst. Rev.* 7:D10783. 10.1002/14651858.CD010783.pub3 PMC8406467 34313331

[B4] Beard J. Officer A. de Carvalho I. Sadana R. Pot A. Michel J. (2016). The World report on ageing and health: a policy framework for healthy ageing. *Lancet* 387 2145–2154. 10.1016/S0140-6736(15)00516-4 26520231 PMC4848186

[B5] Coelho-Júnior H. Trichopoulou A. Panza F. (2021). Cross-sectional and longitudinal associations between adherence to Mediterranean diet with physical performance and cognitive function in older adults: A systematic review and meta-analysis. *Ageing Res. Rev.* 70:101395. 10.1016/j.arr.2021.101395 34153553

[B6] Cooper C. Sommerlad A. Lyketsos C. Livingston G. (2015). Modifiable predictors of dementia in mild cognitive impairment: a systematic review and meta-analysis. *Am. J. Psychiatry* 172 323–334. 10.1176/appi.ajp.2014.14070878 25698435

[B7] Cunnah K. Howe D. Thorpe J. Dunn R. Platt R. White C. (2021). Training people with dementia/cognitive impairment and their carers in the use of web-based supportive technologies (Innovative practice). *Dementia* 20 796–806. 31718279 10.1177/1471301219887592

[B8] Dominguez L. J. Veronese N. Vernuccio L. Catanese G. Inzerillo F. Salemi G. (2021). Nutrition, physical activity, and other lifestyle factors in the prevention of cognitive decline and Dementia. *Nutrients* 13:4080. 10.3390/nu13114080 PMC8624903 34836334

[B9] Fabrigoule C. Letenneur L. Dartigues J. Zarrouk M. Commenges D. Barberger-Gateau P. (1995). Social and leisure activities and risk of dementia: a prospective longitudinal study. *J. Am. Geriatr. Soc.* 43 485–490. 7730528 10.1111/j.1532-5415.1995.tb06093.x

[B10] Fratiglioni L. Wang H. Ericsson K. Maytan M. Winblad B. (2000). Influence of social network on occurrence of dementia: A community-based longitudinal study. *Lancet* 355 1315–1319. 10.1016/S0140-6736(00)02113-9 10776744

[B11] Gerritzen E. Kohl G. Orrell M. McDermott O. (2023). Peer support through video meetings: Experiences of people with young onset dementia. *Dementia* 22 218–234. 10.1177/14713012221140468 36400741 PMC9772896

[B12] Global Burden of Disease Study 2013 Collaborators (2015). Global, regional, and national incidence, prevalence, and years lived with disability for 301 acute and chronic diseases and injuries in 188 countries, 1990-2013: a systematic analysis for the Global Burden of Disease Study 2013. *Lancet* 386 743–800. 26063472 10.1016/S0140-6736(15)60692-4 PMC4561509

[B13] Huang Y. Wang Y. Wang H. Liu Z. Yu X. Yan J. (2019). Prevalence of mental disorders in China: a cross-sectional epidemiological study. *Lancet Psychiatry* 6 211–224. 10.1016/S2215-0366(18)30511-X 30792114

[B14] Jia F. Wang J. Wei N. Sun D. Cao F. (2022). Depression, cognitive reserve markers, and dementia risk in the general population. *Aging Ment. Health* 26 2006–2013. 10.1080/13607863.2021.1972932 34514889

[B15] Jia J. Wang F. Wei C. Zhou A. Jia X. Li F. (2014). The prevalence of dementia in urban and rural areas of China. *Alzheimers Dement.* 10 1–9. 10.1016/j.jalz.2013.01.012 23871765

[B16] Jia L. Quan M. Fu Y. Zhao T. Li Y. Wei C. (2020). Dementia in China: Epidemiology, clinical management, and research advances. *Lancet Neurol.* 19 81–92. 10.1016/S1474-4422(19)30290-X 31494009

[B17] Kim Y. Han S. (2022). Internet use and cognitive functioning in later life: Focus on asymmetric effects and contextual factors. *Gerontologist* 62 425–435. 10.1093/geront/gnab149 34614179 PMC8963164

[B18] Kuiper J. Zuidersma M. Oude Voshaar R. Zuidema S. van den Heuvel E. Stolk R. (2015). Social relationships and risk of dementia: A systematic review and meta-analysis of longitudinal cohort studies. *Ageing Res. Rev.* 22 39–57. 10.1016/j.arr.2015.04.006 25956016

[B19] Lin X. Suanpong K. Ruangkanjanases A. Lim Y. Chen S. (2022). Improving the sustainable usage intention of mobile payments: extended unified theory of acceptance and use of technology model combined with the information system success model and initial trust model. *Front. Psychol.* 12:634911. 10.3389/fpsyg.2021.634911 35082707 PMC8784512

[B20] Liu J. Wang L. Tan J. (2013). Dementia in China: current status. *Neurology* 81 1077–1078. 10.1212/WNL.0b013e3182a4a3cb 24042573

[B21] Livingston G. Huntley J. Sommerlad A. Ames D. Ballard C. Banerjee S. (2020). Dementia prevention, intervention, and care: 2020 report of the Lancet Commission. *Lancet* 396 413–446. 10.1016/S0140-6736(20)30367-6 32738937 PMC7392084

[B22] Lucca U. Tettamanti M. Tiraboschi P. Logroscino G. Landi C. Sacco L. (2020). Incidence of dementia in the oldest-old and its relationship with age: The Monzino 80-plus population-based study. *Alzheimers Dement.* 16 472–481. 10.1016/j.jalz.2019.09.083 31786127

[B23] Pinkston J. Alekseeva N. González Toledo E. (2009). Stroke and dementia. *Neurol. Res.* 31 824–831. 10.1179/016164109X12445505689643 19723451

[B24] Podcasy J. Epperson C. (2016). Considering sex and gender in Alzheimer disease and other dementias. *Dialogues Clin. Neurosci.* 18 437–446. 10.31887/DCNS.2016.18.4/cepperson 28179815 PMC5286729

[B25] Power M. Mormino E. Soldan A. James B. Yu L. Armstrong N. (2018). Combined neuropathological pathways account for age-related risk of dementia. *Ann. Neurol.* 84 10–22. 10.1002/ana.25246 29944741 PMC6119518

[B26] Prince M. Wu F. Guo Y. Gutierrez Robledo L. O’Donnell M. Sullivan R. (2015). The burden of disease in older people and implications for health policy and practice. *Lancet* 385 549–562. 10.1016/S0140-6736(14)61347-7 25468153

[B27] Psaltopoulou T. Sergentanis T. Panagiotakos D. Sergentanis I. Kosti R. Scarmeas N. (2013). Mediterranean diet, stroke, cognitive impairment, and depression: A meta-analysis. *Ann. Neurol.* 74 580–591. 10.1002/ana.23944 23720230

[B28] Qiao H. Du X. Li S. Sun Y. Feng W. Wu Y. (2021). Does older subjective age predict poorer cognitive function and higher risk of dementia in middle-aged and older adults? *Psychiatry Res.* 298 113807. 10.1016/j.psychres.2021.113807 33631534

[B29] Radić B. Blažeković A. Duraković D. Jurišić-Kvesić A. Bilić E. Borovečki F. (2021). Could Mental and Physical Exercise Alleviate Alzheimer’s Disease? *Psychiatr. Danub.* 33 1267–1273. 35503939

[B30] Rehman A. U. Bashir S. Mahmood A. Karim H. Nawaz Z. (2022). Does e-shopping service quality enhance customers’ e-shopping adoption? An extended perspective of unified theory of acceptance and use of technology. *PLoS One* 17:e263652. 10.1371/journal.pone.0263652 35213565 PMC8880925

[B31] Richards M. Hardy R. Wadsworth M. (2003). Does active leisure protect cognition? Evidence from a national birth cohort. *Soc. Sci. Med.* 56 785–792. 10.1016/s0277-9536(02)00075-8 12560011

[B32] Sachdev P. (2022). Social health, social reserve and dementia. *Curr. Opin. Psychiatry* 35 111–117. 10.1097/YCO.0000000000000779 35084381

[B33] Seifert A. (2020). The Digital Exclusion of Older Adults during the COVID-19 Pandemic. *J. Gerontol. Soc. Work.* 63 674–676. 10.1080/01634372.2020.1764687 32401181

[B34] Singh B. Parsaik A. Mielke M. Erwin P. Knopman D. Petersen R. (2014). Association of mediterranean diet with mild cognitive impairment and Alzheimer’s disease: a systematic review and meta-analysis. *J. Alzheimers Dis.* 39 271–282. 10.3233/JAD-130830 24164735 PMC3946820

[B35] Singh N. Sinha N. Liébana-Cabanillas F. J. (2020). Determining factors in the adoption and recommendation of mobile wallet services in India: Analysis of the effect of innovativeness, stress to use and social influence. *Int. J. Inform. Manage.* 50 191–205.

[B36] Sofi F. Abbate R. Gensini G. Casini A. (2010). Accruing evidence on benefits of adherence to the Mediterranean diet on health: An updated systematic review and meta-analysis. *Am. J. Clin. Nutr.* 92 1189–1196. 10.3945/ajcn.2010.29673 20810976

[B37] Spence J. Azarpazhooh M. Larsson S. Bogiatzi C. Hankey G. (2020). Stroke Prevention in Older Adults: Recent Advances. *Stroke* 51 3770–3777. 10.1161/STROKEAHA.120.031707 33121384

[B38] Stephan Y. Sutin A. Luchetti M. Terracciano A. (2017). Feeling older and the development of cognitive impairment and dementia. *J. Gerontol. B Psychol. Sci. Soc. Sci.* 72 966–973. 10.1093/geronb/gbw085 27436103 PMC5927095

[B39] Stern Y. (2002). What is cognitive reserve? Theory and research application of the reserve concept. *J. Int. Neuropsychol. Soc.* 8 448–460. 11939702

[B40] Stute P. Wienges J. Koller A. S. Giese C. Wesemüller W. Janka H. (2021). Cognitive health after menopause: Does menopausal hormone therapy affect it? *Best Pract. Res. Clin. Endocrinol. Metab.* 35 101565. 10.1016/j.beem.2021.101565 34538724

[B41] Wang H. Karp A. Winblad B. Fratiglioni L. (2002). Late-life engagement in social and leisure activities is associated with a decreased risk of dementia: a longitudinal study from the Kungsholmen project. *Am. J. Epidemiol.* 155 1081–1087. 10.1093/aje/155.12.1081 12048221

[B42] Wei N. Sun D. Huang W. (2022). Effects of WeChat use on the subjective health of older adults. *Front. Psychol.* 13:919889. 10.3389/fpsyg.2022.919889 36172240 PMC9511167

[B43] Wilson R. Mendes De Leon C. Barnes L. Schneider J. Bienias J. Evans D. (2002). Participation in cognitively stimulating activities and risk of incident Alzheimer disease. *JAMA* 287 742–748. 10.1001/jama.287.6.742 11851541

[B44] Wilson S. Ardle R. M. Tolley C. Slight S. (2022). Usability and acceptability of wearable technology in the early detection of dementia. *Alzheimers Dement.* 18:e59820. 10.1002/alz.059820 36537475

[B45] Xu H. Zhang Z. Li L. Liu J. (2018). Early life exposure to China’s 1959-61 famine and midlife cognition. *Int. J. Epidemiol.* 47 109–120. 10.1093/ije/dyx222 29126190 PMC6075478

[B46] Yu F. Ryan L. Schaie K. Willis S. Kolanowski A. (2009). Factors associated with cognition in adults: The Seattle longitudinal study. *Res. Nurs. Health* 32 540–550. 10.1002/nur.20340 19606423 PMC2944230

[B47] Zhang M. Y. (1990). The prevalence of dementia and Alzheimer’s disease in Shanghai, China: impact of age, gender, and education. *Ann. Neurol.* 27 428–437. 2353798 10.1002/ana.410270412

[B48] Zhao Y. Hu Y. Smith J. Strauss J. Yang G. (2014). Cohort profile: The China Health and Retirement Longitudinal Study (CHARLS). *Int. J. Epidemiol.* 43 61–68. 10.1093/ije/dys203 23243115 PMC3937970

[B49] Zhao Y. Strauss J. Chen X. Wang Y. Gong J. Meng Q. (2020). *China Health and Retirement Longitudinal Study Wave 4 User’s Guide.* Beijing: Peking University.

[B50] Zhu Y. Liu H. Lu X. Zhang B. Weng W. Yang J. (2019). Prevalence of dementia in the People’s Republic of China from 1985 to 2015: A systematic review and meta-regression analysis. *BMC Public Health* 19:578. 10.1186/s12889-019-6840-z 31092218 PMC6521412

