# Supplementary material for: Relationship between digital exclusion and cognitive impairment in Chinese adults
Source: Front Aging Neurosci. 2023 Jul 3;15:1194348. doi: 10.3389/fnagi.2023.1194348 (PMC10350515; doi:10.3389/fnagi.2023.1194348)
Supplement: Supplementary file 1 [file Table_1.DOC]

Supplementary 1. Multivariate analysis of the associations between using WeChat, mobile payments and cognitive impairment, in the overall population and sub-population by age (Age <80 years old, Age <70 years old).

| **Variables** | **Overall population** | | **Age <80 years old** | | **Age <70 years old** | |
| --- | --- | --- | --- | --- | --- | --- |
|  | *OR* [95%CI] | *P* | *OR* [95%CI] | *P* | *OR* [95%CI] | *P* |
| Digital exclusion |  |  |  |  |  |  |
| Neither WeChat nor mobile payments | 3.48(2.27, 5.33) | <0.001 | 3.25 (2.12, 4.98) | <0.001 | 2.52 (1.63, 3.90) | <0.001 |
| Only WeChat | 1.86(1.10, 3.16) | 0.021 | 1.77 (1.04, 3.00) | 0.035 | 1.42 (0.82, 2.48) | 0.212 |
| Use WeChat and mobile payments | Ref. | - | Ref. | - | Ref. | - |
| Age | 1.09(1.08, 1.10) | <0.001 | 1.10(1.09,1.11) | <0.001 | 1.18(1.16, 1.20) | <0.001 |
| Sex |  |  |  |  |  |  |
| Female | Ref. | - | Ref. | - | Ref. | - |
| Male | 0.79(0.65, 0.96) | 0.015 | 0.86(0.70, 1.04) | 0.121 | 0.90(0.70, 1.15) | 0.386 |
| Education |  |  |  |  |  |  |
| Junior high and below | 1.12(0.93, 1.35) | 0.242 | 1.07(0.89, 1.30) | 0.468 | 1.00(0.80, 1.25) | 0.992 |
| High school and above | Ref. | - | Ref. | - | Ref. | - |
| Smoking status |  |  |  |  |  |  |
| Still have | 1.43(1.17, 1.74) | <0.001 | 1.33(1.08,1.62) | 0.006 | 1.34(1.05, 1.71) | 0.018 |
| Quit | 1.16(0.94, 1.44) | 0.174 | 1.13(0.90, 1.41) | 0.282 | 1.19(0.90, 1.56) | 0.223 |
| Never smoked | Ref. | - | Ref. | - | Ref. | - |
| Drinking condition |  |  |  |  |  |  |
| Drink more than once a month | 0.93(0.80, 1.09) | 0.384 | 0.90(0.76, 1.05) | 0.006 | 0.85(0.71, 1.04) | 0.108 |
| Drink but less than once a month | 0.86(0.67, 1.11) | 0.244 | 0.85(0.66, 1.11) | 0.232 | 0.73(0.53, 1.00) | 0.047 |
| None of these | Ref. | - | Ref. | - | Ref. | - |
| Living alone |  |  |  |  |  |  |
| Yes | 1.17(0.98, 1.40) | 0.082 | 1.18(0.98, 1.42) | 0.080 | 1.16(0.91, 1.48) | 0.233 |
| No | Ref. | - | Ref. | - | Ref. | - |
| Physical activities |  |  |  |  |  |  |
| Yes | 0.90(0.72, 1.14) | 0.384 | 0.92(0.73, 1.17) | 0.506 | 0.82(0.61, 1.11) | 0.201 |
| No | Ref. | - | Ref. | - | Ref. | - |
| Social activities |  |  |  |  |  |  |
| Yes | 0.83(0.73, 0.95) | 0.005 | 0.84(0.73, 0.96) | 0.009 | 0.81(0.69, 0.96) | 0.012 |
| No | Ref. | - | Ref. | - | Ref. | - |
| Hypertension |  |  |  |  |  |  |
| Yes | 1.12(0.92, 1.37) | 0.270 | 1.13(0.92, 1.38) | 0.243 | 1.08(0.85, 1.38) | 0.519 |
| No | Ref. | - | Ref. | - | Ref. | - |
| Diabetes |  |  |  |  |  |  |
| Yes | 0.97(0.73, 1.30) | 0.847 | 0.96(0.72, 1.29) | 0.803 | 0.87(0.62, 1.24) | 0.446 |
| No | Ref. | - | Ref. | - | Ref. | - |
| Dyslipidemia |  |  |  |  |  |  |
| Yes | 0.98(0.79, 1.21) | 0.868 | 0.98(0.79, 1.22) | 0.873 | 1.01(0.79, 1.30) | 0.928 |
| No | Ref. | - | Ref. | - | Ref. | - |
| Stroke |  |  |  |  |  |  |
| Yes | 1.32(1.02, 1.70) | 0.036 | 1.30(1.00, 1.69) | 0.052 | 1.52(1.11, 2.07) | 0.009 |
| No | Ref. | - | Ref. | - | Ref. | - |
| Chronic lung diseases |  |  |  |  |  |  |
| Yes | 1.06(0.80, 1.39) | 0.693 | 1.04(0.79, 1.38) | 0.772 | 0.81(0.57, 1.15) | 0.232 |
| No | Ref. | - | Ref. | - | Ref. | - |
| Emotional, nervous, or psychiatric problems |  |  |  |  |  |  |
| Yes | 0.64(0.28, 1.44) | 0.279 | 0.66(0.29, 1.49) | 0.317 | 0.44(0.15, 1.27) | 0.128 |
| No | Ref. | - | Ref. | - | Ref. | - |
| Memory-related disease |  |  |  |  |  |  |
| Yes | 1.48(0.99, 2.22) | 0.058 | 1.66(1.09, 2.52) | 0.017 | 1.75(1.03, 2.95) | 0.038 |
| No | Ref. | - | Ref. | - | Ref. | - |
| Hearing disorder |  |  |  |  |  |  |
| Yes | 1.14(0.95, 1.37) | 0.156 | 1.20(1.00, 1.45) | 0.054 | 1.20(0.94, 1.51) | 0.137 |
| No | Ref. | - | Ref. | - | Ref. | - |

Multivariate analysis: Adjusted for age, sex, education level, smoking status, drinking condition, living alone, physical activities, social activities, hypertension, diabetes, dyslipidemia, stroke, chronic lung diseases, emotional, nervous, or psychiatric problems, memory-related disease, hearing disorder

**Supplementary 2. Multivariate analysis of the ssociations between using WeChat, mobile payments and cognitive impairment, in the sub-population by residential status (urban, transitional zone, rural).**

| **Variables** | **Urban** | | **Transitional zone between urban and rural areas** | | **Rural** | |
| --- | --- | --- | --- | --- | --- | --- |
|  | *OR* [95%CI] | *P* | *OR* [95%CI] | *P* | *OR* [95%CI] | *P* |
| Digital exclusion |  |  |  |  |  |  |
| Neither WeChat nor mobile payments | 3.44 (1.73, 6.84) | <0.001 | 3.64 (1.17, 11.34) | 0.026 | 2.91 (1.52, 5.58) | 0.001 |
| Only WeChat | 2.76 (1.24, 6.14) | 0.012 | 2.93 (0.81, 10.65) | 0.102 | 1.08 (0.45, 2.59) | 0.866 |
| Use WeChat and mobile payments | Ref. | - | Ref. | - | Ref. | - |
| Age | 1.07(1.05, 1.08) | <0.001 | 1.09(1.06, 1.13) | <0.001 | 1.10(1.09, 1.11) | <0.001 |
| Sex |  |  |  |  |  |  |
| Female | Ref. | - | Ref. | - | Ref. | - |
| Male | 0.69(0.44, 1.08) | 0.105 | 1.01(0.48, 2.15) | 0.975 | 0.75(0.60, 0.94) | 0.012 |
| Education |  |  |  |  |  |  |
| Junior high and below | 1.16(0.83, 1.62) | 0.395 | 0.70(0.38, 1.28) | 0.250 | 0.83(0.64, 1.08) | 0.169 |
| High school and above | Ref. | - | Ref. | - | Ref. | - |
| Smoking status |  |  |  |  |  |  |
| Still have | 1.85(1.16, 2.96) | 0.010 | 2.70(1.29, 5.66) | 0.009 | 1.23(0.98, 1.54) | 0.079 |
| Quit | 1.60(0.96, 2.65) | 0.070 | 1.02(0.44, 2.40) | 0.958 | 1.10(0.86, 1.42) | 0.449 |
| Never smoked | Ref. | - | Ref. | - | Ref. | - |
| Drinking condition |  |  |  |  |  |  |
| Drink more than once a month | 0.94(0.64, 1.38) | 0.744 | 1.19(0.62, 2.31) | 0.597 | 0.90(0.75, 1.07) | 0.236 |
| Drink but less than once a month | 0.93(0.54, 1.62) | 0.800 | 2.11(0.97, 4.58) | 0.060 | 0.75(0.55, 1.03) | 0.074 |
| None of these | Ref. | - | Ref. | - | Ref. | - |
| Living alone |  |  |  |  |  |  |
| Yes | 1.54(1.06, 2.25) | 0.025 | 0.97(0.43, 2.17) | 0.939 | 1.17(0.94, 1.44) | 0.154 |
| No | Ref. | - | Ref. | - | Ref. | - |
| Physical activities |  |  |  |  |  |  |
| Yes | 0.70(0.41, 1.20) | 0.200 | 1.02(0.33, 3.15) | 0.978 | 1.00(0.77, 1.30) | 0.990 |
| No | Ref. | - | Ref. | - | Ref. | - |
| Social activities |  |  |  |  |  |  |
| Yes | 0.92(0.67, 1.28) | 0.632 | 1.70(0.92, 3.14) | 0.090 | 0.80(0.69, 0.93) | 0.004 |
| No | Ref. | - | Ref. | - | Ref. | - |
| Hypertension |  |  |  |  |  |  |
| Yes | 1.06(0.66, 1.71) | 0.815 | 0.80(0.33,1.94) | 0.629 | 1.13(0.90, 1.43) | 0.290 |
| No | Ref. | - | Ref. | - | Ref. | - |
| Diabetes |  |  |  |  |  |  |
| Yes | 1.10(0.60, 2.01) | 0.768 | 0.85(0.28, 2.61) | 0.783 | 0.98(0.69, 1.38) | 0.903 |
| No | Ref. | - | Ref. | - | Ref. | - |
| Dyslipidemia |  |  |  |  |  |  |
| Yes | 0.93(0.59, 1.47) | 0.765 | 0.98(0.45, 2.12) | 0.957 | 1.06(.82, 1.37) | 0.657 |
| No | Ref. | - | Ref. | - | Ref. | - |
| Stroke |  |  |  |  |  |  |
| Yes | 1.45(0.84, 2.49) | 0.180 | 0.96(0.33, 2.76) | 0.938 | 1.32(0.97, 1.79) | 0.081 |
| No | Ref. | - | Ref. | - | Ref. | - |
| Chronic lung diseases |  |  |  |  |  |  |
| Yes | 1.11(0.58, 2.13) | 0.754 | 2.73(1.10, 6.81) | 0.031 | 0.95(0.69, 1.32) | 0.777 |
| No | Ref. | - | Ref. | - | Ref. | - |
| Emotional, nervous, or psychiatric problems |  |  |  |  |  |  |
| Yes | 1.00 | - | 1.00 | - | 0.82(0.35, 1.92) | 0.645 |
| No | Ref. | - | Ref. | - | Ref. | - |
| Memory-related disease |  |  |  |  |  |  |
| Yes | 0.72(0.23, 2.24) | 0.575 | 1.81(0.33, 9.91) | 0.497 | 1.66(1.05, 2.65) | 0.031 |
| No | Ref. | - | Ref. | - | Ref. | - |
| Hearing disorder |  |  |  |  |  |  |
| Yes | 1.21(0.77, 1.90) | 0.419 | 1.43(0.61, 3.38) | 0.415 | 1.07(0.87, 1.32) | 0.513 |
| No | Ref. | - | Ref. | - | Ref. | - |

Multivariate analysis: Adjusted for age, sex, education level, smoking status, drinking condition, living alone, physical activities, social activities, hypertension, diabetes, dyslipidemia, stroke, chronic lung diseases, emotional, nervous, or psychiatric problems, memory-related disease, hearing disorder
